# Lesser Toxicities of Belotecan in Patients with Small Cell Lung Cancer: A Retrospective Single-Center Study of Camptothecin Analogs

**DOI:** 10.1155/2016/3576201

**Published:** 2016-11-27

**Authors:** Yeon Hee Park, Chae Uk Chung, Bo Mi Park, Myoung Rin Park, Dong Il Park, Jae Young Moon, Hee Sun Park, Jin Hwan Kim, Sung Soo Jung, Ju Ock Kim, Sun Young Kim, Jeong Eun Lee

**Affiliations:** ^1^Department of Internal Medicine, College of Medicine, Chungnam National University, Daejeon, Republic of Korea; ^2^Department of Internal Medicine, College of Medicine, Eulji University, Daejeon, Republic of Korea; ^3^Department of Radiology, College of Medicine, Chungnam National University, Daejeon, Republic of Korea

## Abstract

*Purpose*. Topotecan and belotecan are camptothecin derivatives that are used to treat small cell lung cancer (SCLC). This study compared the toxicities and efficacies of belotecan and topotecan monotherapies in patients with SCLC.* Methods*. We retrospectively reviewed data from 94 patients with SCLC (with or without prior chemotherapy) who were treated using belotecan monotherapy (*n* = 59, 188 cycles) or topotecan monotherapy (*n* = 35, 65 cycles) between September 2003 and December 2011.* Results*. Thrombocytopenia occurred during 42% and 61.5% of the belotecan and topotecan cycles, respectively (*p* = 0.007). Significant differences between belotecan and topotecan were also observed for grade 4/5 lung infection (3.2% versus 10.8%, resp.; *p* = 0.003), all-grade headache (3.2% versus 10.8%, resp.; *p* = 0.017), and grade 4/5 increased liver enzymes (0.5% versus 4.6%, resp.; *p* = 0.023). The median TTPDs, CSSs, and OSs were 14 months and 11.6 months (*p* = 0.646), 10 months and 7 months (*p* = 0.179), and 34.5 months and 21.4 months (*p* = 0.914) after belotecan and topotecan monotherapy, respectively.* Conclusions*. Belotecan monotherapy may be safer than topotecan monotherapy in SCLC patients. And in terms of efficacy, belotecan could be comparable to topotecan monotherapy.

## 1. Introduction

Small cell lung cancer (SCLC) accounts for approximately 15% of all lung cancer cases in United States [1–3]. And in South Korea, it also accounts for 12.5% of lung cancer in 2012 [4, 5]. Unfortunately, treatment is not effective in altering the high recurrence rate and short survival, regardless of the initial stage of SCLC at the diagnosis [6, 7]. Therefore, combination chemotherapy (e.g., etoposide/cisplatin) remains the main treatment for patients with extensive-stage SCLC [8–12]. The etoposide/cisplatin combination provides initial response rates of up to 80%, although most patients ultimately relapse and receive second-line therapy [13, 14]. However, there are only a few chemotherapeutic drugs that can be used as second-line therapy for SCLC. Various single-agent treatments (e.g., topotecan, belotecan, etoposide, irinotecan, gemcitabine, and pemetrexed) have been approved as second-line chemotherapy for patients with SCLC. The response rates for single-agent treatments are 0–47% [15]. As there are few effective treatments for SCLC, additional safe and effective treatments are urgently needed.

Topotecan is approved as chemotherapeutic drug for relapsed or failed after first-line therapy of SCLC. It is also available as intravenous and oral agent. Topotecan showed greater response rate than combination of cyclophosphamide, doxorubicin, and vincristine regimen, although median survival was similar [16]. In a randomized controlled trial, oral topotecan was superior to best supportive care in improving survival and quality of life [17]. As second-line therapy, topotecan demonstrated response rate of 10~40% and median survival time of 6.0 months [18].

Belotecan (Camtobell®, CKD602, 7-[2-(N-isopropylamino)ethyl]-(20S)-camptothecin, Chong Keun Dang Corp.) is a novel camptothecin derivative that has a water-solubilizing chemical group attached to the B ring. There are few studies that have compared the clinical effects of belotecan and topotecan monotherapies in patients with SCLC. Therefore, this study aimed to assess and compare toxicities and efficacies between belotecan and topotecan monotherapies in patients with SCLC.

## 2. Patients and Methods

This retrospective study evaluated patients with SCLC who had received belotecan or topotecan monotherapy. The study's design was approved by the institutional review board of Chungnam National University Hospital (2013-01-008). All patients were informed that they were eligible to receive belotecan or topotecan monotherapy, and their consent to receive treatment was recorded in their medical records.

### 2.1. Eligibility Criteria

All data were derived from a database of patients who were diagnosed with SCLC between September 2003 and December 2011. The inclusion criteria were histological or cytological confirmation of SCLC, treatment with belotecan or topotecan monotherapy for SCLC (first-line, second-line, or third-line), no prior chemotherapy using an topoisomerase I inhibitor, no other malignant disease, no uncontrolled disease that might have affected the patient's survival, and no chemotherapy within 3 weeks of the study period. The laboratory criteria were white blood cell counts of ≥3,000/mm^3^, absolute neutrophil counts of ≥1,000/mm^3^, platelet counts of ≥100,000/mm^3^, hemoglobin levels of ≥10.0 g/dL, serum bilirubin levels of ≤1.8 mg/dL, serum transaminase levels of ≤100 IU/L, and serum creatinine levels of ≤1.5 mg/dL.

### 2.2. Chemotherapy

All patients in the present study received belotecan or topotecan monotherapy as first-line, second-line, or third-line therapy. All patients were required to wait at least 3 weeks after their last cycle before undergoing second- or third-line therapy. Belotecan monotherapy was administered at 0.5 mg/m^2^/day for 5 consecutive days every 3 weeks. The belotecan was mixed with 100 mL of 5% dextrose and administered as a 30 min intravenous injection. Topotecan monotherapy was administered at 1.5 mg/m^2^/day for 5 consecutive days every 3 weeks. The topotecan was mixed with 200 mL of 5% dextrose and administered as a 30 min intravenous injection. Dose adjustments were made at the start of each new cycle and were made based on the worst toxicity that was observed during the previous cycle. If the patient experienced grade 4/5 toxicities, the topotecan dose reduction was 0.25 mg/m^2^ or a 20% reduction from the previous dose of belotecan. Belotecan or topotecan monotherapy was terminated when the treatment response revealed progressive disease or chemotherapy-induced toxicity that was uncontrolled by consecutive dose reductions or if the treatment schedule was delayed by >2 weeks.

### 2.3. Response and Toxicity Evaluation

Tumor response was evaluated using the Response Evaluation Criteria in Solid Tumours (RECIST, version 1.1) via enhanced computed tomography. Hematological and nonhematological toxicities were evaluated using the Common Terminology Criteria for Adverse Events (version 4.0).

### 2.4. Survival Analysis

Time to progressive disease (TTPD) was defined as the time from the beginning of belotecan or topotecan monotherapy to the date of diagnosing radiologically confirmed progressive disease (PD). Chemotherapy-specific survival (CSS) was defined as the time from the beginning of belotecan or topotecan monotherapy to the date of cancer-related death or the end of the study. Overall survival (OS) was defined as the time from the date of histological or pathological confirmation to the date of cancer-related death or the end of the study.

### 2.5. Statistical Analysis

We used the chi-square and independent *t*-tests to analyze the differences in the patients' baseline characteristics and toxicities. We also used the chi-square test and Fisher's exact test to analyze the differences in toxicities between patients who were <75 years old and ≥75 years old. The TTPD, CSS, and OS values for the two chemotherapy groups were evaluated using the Kaplan-Meier method and the log-rank test. All statistical analyses were performed using SPSS software (version 19; SPSS Inc., Chicago, IL), and a *p* value of <0.05 was considered statistically significant.

## 3. Results

### 3.1. Baseline Characteristics of the Two Treatment Groups

Between September 2003 and November 2011, we identified 94 patients who have received topotecan monotherapy (*n* = 35) or belotecan monotherapy (*n* = 59) (Table 1). The median patient age was 68 years (range, 42–88 years), and there were no significant differences between the two treatment groups, except in their previous lines of chemotherapy. Most treatments for all patients were second-line (60.6%) and third-line (25.5%) therapy, respectively. Most patients in the belotecan group received belotecan as second-line therapy (67.8%). In topotecan group, the proportion of patients who received as second-line was 48.6% and as third-line was 42.9%.

### 3.2. Tumor Response

Among the 94 included patients, 28 patients could not be evaluated for response, due to inadequate radiological data (Table 2). Fifteen of these unevaluable patients were treated using topotecan and 13 patients were treated using belotecan. Among the 46 evaluable patients who received belotecan, the best overall responses were complete response (CR) in 2 patients (4.3%), partial response (PR) in 8 patients (17.4%), stable disease (SD) in 19 patients (41.3%), and PD in 17 patients (37%). The overall response rate (ORR) for belotecan was 21.7% in the intent-to-treat analysis. Among the 20 evaluable patients who received topotecan, the best overall responses were PR in 1 patient (5%), SD in 7 patients (35%), and PD in 12 patients (60%). The ORR in the topotecan group was 5%, which was noticeably lower than that in the belotecan group.

### 3.3. Toxicity

The toxicity analyses for each group were based on the total number of treatment cycles. The belotecan group contained 59 patients who underwent 188 cycles (mean 3.19 cycles), and the topotecan group contained 35 patients who underwent 65 cycles (mean 1.86 cycles) (Table 3). Among the hematological toxicities, all-grade thrombocytopenia and grade 4/5 thrombocytopenia were significantly more common in the topotecan group, compared to the belotecan group (*p* = 0.007 and *p* = 0.001, resp.). There were no statistically significant differences in the frequencies of anemia and neutropenia, although all-grade anemia was slightly more frequent in the belotecan group, and all-grade neutropenia was slightly more frequent in the topotecan group. We also evaluated a wide variety of nonhematological toxicities. Compared to the belotecan group, the topotecan group exhibited significantly more frequent grade 4/5 lung infection (*p* = 0.003), grade 4/5 increased liver enzymes (*p* = 0.23), and all-grade headache (*p* = 0.017).

### 3.4. Comparing Toxicities according to Age

The toxicities were also compared for patients who were <75 years old and ≥75 years old (Table 4). Among patients who received belotecan monotherapy, neutropenia was significantly more frequent for patients who were ≥75 years old (77% versus 58%, *p* = 0.012). Among the nonhematological toxicities, patients who were ≥75 years old exhibited significantly higher frequencies of skin rash (6.3% versus 0.8%, *p* = 0.047) and constipation (7.8% versus 1.6%, *p* = 0.046). However, there were no age-related differences in the toxicities among the patients who received topotecan monotherapy.

### 3.5. Survival Analysis

For the belotecan group, the median TTPD was 14.0 months, the median CSS was 10.0 months, and the median OS was 34.5 months. For the topotecan group, the median TTPD was 11.6 months, the median CSS was 7.0 months, and the median OS was 21.4 months (see Supplemental Figure  1 available online at http://dx.doi.org/10.1155/2016/3576201). There were no significant differences in TTPD, CSS, and OS between the two treatment groups (*p* = 0.646, *p* = 0.179, and *p* = 0.914, resp.).

## 4. Discussion

In the present study, we observed that belotecan monotherapy and topotecan monotherapy had comparable efficacies in SCLC. However, compared to topotecan monotherapy, belotecan monotherapy was associated with significantly less frequent thrombocytopenia (all-grade and grade 4/5), grade 4/5 lung infection, and all-grade headache. Therefore, we cautiously speculate that belotecan may be preferable to topotecan, given the similar survival outcomes and superior safety profile of belotecan.

In a tumor xenograft model, belotecan provided an approximately 3-fold more potent antitumor effect, compared to topotecan. Furthermore, the therapeutic margin for belotecan was 4-fold higher than that for topotecan [19]. In other words, belotecan could be more safely used than topotecan.

Topotecan and belotecan monotherapies are both second-line options for SCLC; however, only two studies had compared the survival rates and toxicities of topotecan and belotecan in patients with SCLC and both were reported as abstracts [20]. Yoon et al. compared belotecan and topotecan as second-line treatment in SCLC patients. ORR, PFS, and OS of belotecan and topotecan were not significant different. However, grade 3/4 neutropenia was more common in topotecan group (43.6%) than belotecan group (21.3%) (*p* = 0.016). A prospective phase III study comparing belotecan and topotecan was also reported at the 112th Annual Meeting of Korean Academy of Tuberculosis and Respiratory Diseases (in 2011) [21]. They compared the efficacies and hematological toxicities of belotecan and topotecan in 54 patients with relapsed SCLC after receiving platinum/etoposide combination therapy. Compared to belotecan monotherapy, topotecan monotherapy was associated with more frequent grade 3/4 hematological toxicity (64.3% versus 96.2%, resp.), although the two groups exhibited similar efficacy outcomes (including ORR, disease control rate, OS, and progression-free survival). Those findings are also consistent with the findings of the present study.

We compared two phase II trials of belotecan and topotecan which included previously untreated extensive-stage SCLC [22, 23]. Belotecan study showed superior efficacy in ORR (53.2% versus 39.0%) and 1 year-survival rate (49.9% versus 39%), although TTPD and OS were similar in both studies. Interestingly, grade 3/4 neutropenia and thrombocytopenia were 71.0% and 12.9% in belotecan study. However, in topotecan study, grade 3/4 neutropenia and thrombocytopenia were 92% and 38%. These data also support our result that belotecan therapy was better in safety profile than topotecan therapy.

Another study compared the efficacies and toxicities of topotecan- and belotecan-based chemotherapies for recurrent epithelial ovarian cancer. In that study, belotecan monotherapy provided a superior safety profiles compared to topotecan monotherapy. Grade 3/4 anemia and thrombocytopenia were less frequent during belotecan-based chemotherapy [24].

In this study, belotecan had greater response rate and survival rate than topotecan. However, it was not statistically significant. These results would be influenced by different proportion of patients between two groups. In topotecan group, patients who were treated as third line were 42.9%; however, 15.3% of belotecan group were treated as third line. Furthermore, topotecan group had been performed fewer cycles than belotecan group (mean 1.86 versus 3.19 cycles). This also would have impact on efficacy outcome.

Moreover, older patient group (who were ≥75 years old) had more adverse events than younger patient group (who were <75 years old) when treated by belotecan. Therefore, we should be cautious when treating elderly patients with belotecan therapy.

### 4.1. Limitations

The first limitation of the present study is its retrospective design. The second limitation is the fact that we cannot account for the effects of previous treatment(s). For example, belotecan was most frequently used as a second-line therapy (*n* = 40, 67.8%), which was followed by first-line therapy (*n* = 10, 16.9%). Topotecan was also most frequently used as a second-line therapy (*n* = 17, 48.6%), although this was followed by third-line therapy (*n* = 15, 42.9%), and these differences were statistically significant. Therefore, we cannot overlook the possible effects of the previous chemotherapeutic regimens. Nevertheless, all subsequent lines of therapy were delayed until the patients' symptoms had improved and their laboratory findings were normal, which may have prevented any cumulative toxicity. The third limitation is that we could not clearly distinguish the nonhematological toxicities (e.g., headache and dyspnea) from the symptoms of lung cancer. The fourth limitation is that response for 29.8% of all patients were not evaluated. For that reason, we could not compare survival and response rate precisely. Thus, further prospective studies are needed to evaluate the efficacies and toxicities of belotecan and topotecan monotherapies, in order to develop more potent and well-tolerated chemotherapies.

## 5. Conclusion

In the present study, hematological toxicities were generally more frequent among patients who received topotecan monotherapy, and a significant difference was observed for grade 4/5 thrombocytopenia. A similar trend was observed for nonhematological toxicities, with significant differences being observed for all-grade headache, grade 4/5 increased liver enzymes, and grade 4/5 lung infection. Therefore, based on our experience, belotecan monotherapy may be safer than topotecan monotherapy in SCLC and it also showed comparable efficacies.

## Supplementary Material

Kaplan-Meier survival analysis

## Figures and Tables

**Table 1:** Baseline characteristics.

Parameter	All patients (*n* = 94)		Belotecan group (*n* = 59)		Topotecan group (*n* = 35)		*p* value
	Number of patients	%	Number of patients	%	Number of patients	%	
*Sex*							0.054
Male	80	85.1	47	79.7	33	94.3	
Female	14	14.9	12	20.3	2	5.7	
*Age, years*							0.164
Median	67.8		68.8		65.8		
Range	42–88		42–88		48–81		
*ECOG *							0.715
0	17	18.1	10	16.9	7	20	
1	48	51.1	31	52.5	17	48.6	
2	18	19.1	11	18.6	7	20	
3	10	10.6	7	11.9	3	8.6	
4	1	1.1	0	0	1	2.9	
*Stage at diagnosis*							0.330
Limited	44	46.8	32	54.2	12	34.3	
IIa	2	2.1	2	3.4	0	0	
IIb	1	1.1	1	1.7	0	0	
IIIa	15	16	11	18.6	4	11.4	
IIIB	26	27.7	18	30.5	8	22.9	
Extensive	50	53.2	27	45.8	23	65.7	
*Response to prior CTx*							0.088
CR or PR	11	11.7	10	16.9	1	2.9	
SD or PD	55	58.5	36	61	19	54.3	
*CTx line *							0.011
1	13	13.8	10	16.9	3	8.6	
2	57	60.6	40	67.8	17	48.6	
3	24	25.5	9	15.3	15	42.9	

ECOG: Eastern Cooperative Oncology Group score, CTx: chemotherapy, CR: complete response, PR: partial response, SD: stable disease, and PD: progressive disease.

**Table 2:** Chemotherapy response.

	All patients (*n* = 94)		Belotecan (*n* = 59)		Topotecan (*n* = 35)	
	Number	%	Number	%	Number	%
Complete response	2/66	3.0	2/46	4.3	0	0
Partial response	9/66	13.6	8/46	17.4	1/20	5
Stable disease	26/66	26.3	19/46	41.3	7/20	35
Progressive disease	29/66	43.9	17/46	37	12/20	60
Not evaluated	28/94	29.8	13/59	22	15/35	42.9
Response rate	11/66	16.7	10/46	21.7	1/20	5

Response rate = complete response + partial response.

**Table 3:** Toxicities (CTCAE version 4.0).

CTCAE toxicity	Belotecan (*n* = 188)				Topotecan (*n* = 65)				All grades *p* value	Grade 4/5 *p* value
	All grades		Grade 4/5		All grades		Grade 4/5			
	Number	%	Number	%	Number	%	Number	%		
*Hematological toxicity*										
Anemia	125	66.5	3	1.6	42	64.6	0	0	0.783	0.306
Neutropenia	120	63.8	56	29.8	47	72.3	24	36.9	0.214	0.286
Thrombocytopenia	79	42	12	6.4	40	61.5	14	21.5	**0.007**	**0.001**
*Nonhematological toxicity*										
Prurigo nodularis	0	0	0	0	1	1.5	0	0	0.088	
Generalized muscle weakness	22	11.7	0	0	10	15.4	0	0	0.441	
Anorexia	24	12.8	0	0	9	13.8	0	0	0.824	
Constipation	7	3.7	0	0	6	9.2	0	0	0.083	
Nausea	15	8	0	0	5	7.7	0	0	0.941	
Vomiting	5	2.7	0	0	2	3.1	0	0	0.860	
Dyspnea	4	2.1	0	0	4	6.2	1	1.5	0.110	0.088
Lung infection	12	6.4	4	2.1	9	13.8	7	10.8	0.060	**0.003**
Dizziness	6	3.2	0	0	5	7.7	0	0	0.125	
Myalgia	4	2.1	0	0	0	0	0	0	0.236	
Sinus tachycardia	0	0	0	0	1	1.5	0	0	0.088	
Headache	6	3.2	0	0	7	10.8	0	0	**0.017**	
Cough	1	0.5	0	0	0	0	0	0	0.556	
Diarrhea	8	4.3	0	0	6	9.2	0	0	0.130	
Skin rash	5	2.7	0	0	2	3.1	0	0	0.860	
Intracranial hemorrhage	1	0.5	1	0.5	0	0	0	0	0.556	0.556
TB increased	0	0	0	0	1	1.5	0	0	0.088	
AST/ALT increased	3	1.6	1	0.5	3	4.6	3	4.6	0.168	**0.023**
Abdominal pain	3	1.6	0	0	1	1.5	0	0	0.975	
Hiccups	7	3.7	0	0	1	1.5	0	0	0.386	
Fever	7	3.7	0	0	1	1.5	0	0	0.386	
Confusion	0	0	0	0	1	1.5	0	0	0.088	
Hyponatremia	3	1.6	1	0.5	2	3.1	1	1.5	0.460	0.430
Delirium	0	0	0	0	1	1.5	0	0	0.088	
Insomnia	2	1.1	0	0	0	0	0	0	0.404	
Tremor	1	0.5	0	0	0	0	0	0	0.556	
Sore throat	3	1.6	0	0	0	0	0	0	0.306	
URI	1	0.5	0	0	0	0	0	0	0.556	
Viral hepatitis	1	0.5	1	0.5	0	0	0	0	0.556	0.556
Dyspepsia	0	0	0	0	1	1.5	0	0	0.088	
Ileus	0	0	0	0	1	1.5	0	0	0.088	
Atrial fibrillation	1	0.5	0	0	0	0	0	0	0.556	
Hypokalemia	1	0.5	0	0	0	0	0	0	0.556	
Pleural infection	1	0.5	0	0	1	1.5	0	0	0.430	

CTCAE: Common Terminology Criteria for Adverse Events, TB: total bilirubin, ALT: alanine transaminase, AST: aspartate transaminase, and URI: upper respiratory tract infection.

**Table 4:** Comparing the toxicities for patients who were <75 years or ≥75 years old.

CTCAE terminology	Belotecan (188 cycles)				*p* value	Topotecan (65 cycles)				*p* value
	<75 years (124 cycles)		≥75 years (64 cycles)			<75 years (53 cycles)		≥75 years (12 cycles)		
	Number	%	Number	%		Number	%	Number	%	
*Hematological toxicity*										
Anemia	82	66.1	42	65.6	0.945	35	66	7	58.3	0.741
Neutropenia	72	58.1	49	76.6	**0.012**	38	71.7	9	75	1.000
Thrombocytopenia	55	44.4	23	35.9	0.267	33	62.3	7	58.3	1.000
*Nonhematological toxicity*										
Generalized muscle weakness	11	8.9	11	17.2	0.093	10	18.9	0	0	
Anorexia	15	12.1	9	14.1	0.702	9	17	0	0	
Constipation	2	1.6	5	7.8	**0.046**	4	7.5	2	16.7	0.305
Nausea	12	9.7	3	4.7	0.232	5	9.4	0	0	
Vomiting	4	3.2	1	1.6	0.663	2	3.8	0	0	
Dyspnea	1	0.8	3	4.7	0.115	3	5.7	0	0	
Lung infection	8	6.5	3	4.7	0.752	7	13.2	2	16.7	0.667
Dizziness	5	4.0	1	1.6	0.666	5	9.4	0	0	
Myalgia	2	1.6	2	3.1	0.606	0	0	0	0	
Sinus tachycardia	0	0	0	0		1	1.9	0	0	
Headache	6	4.8	0	0		7	13.2	0	0	
Cough	1	0.8	0	0		0	0	0	0	
Diarrhea	3	2.4	5	7.8	0.124	3	5.7	3	25	0.071
Skin rash	1	0.8	4	6.3	**0.047**	2	3.8	0	0	
Intracranial hemorrhage	1	0.8	0	0		0	0	0	0	
TB increased	0	0	0	0		1	1.9	0	0	
AST/ALT increased	2	1.6	1	1.6	1.000	3	5.7	0	0	
Abdominal pain	3	2.4	0	0		1	1.9	0	0	
Hiccups	6	4.8	1	1.6	0.426	1	1.9	0	0	
Fever	5	4.0	2	3.1	1.000	1	1.9	0	0	
Confusion	0	0	0	0		1	1.9	0	0	
Hyponatremia	3	2.4	0	0		2	3.8	0	0	
Delirium	0	0	0	0		1	1.9	0	0	
Insomnia	2	1.6	0	0		0	0	0	0	
Tremor	1	0.8	0	0		0	0	0	0	
Sore throat	2	1.6	1	1.6	1.000	0	0	0	0	
URI	0	0	1	1.6		0	0	0	0	
Viral hepatitis	0	0	1	1.6		0	0	0	0	
Dyspepsia	0	0	0	0		1	1.9	0	0	
Ileus	0	0	0	0		1	1.9	0	0	
Atrial fibrillation	1	0.8	0	0		0	0	0	0	
Hypokalemia	1	0.8	0	0		0	0	0	0	
Pleural infection	1	0.8	0	0		1	1.9	0	0	

CTCAE: Common Terminology Criteria for Adverse Events, TB: total bilirubin, ALT: alanine transaminase, AST: aspartate transaminase, and URI: upper respiratory tract infection.

## Abbreviations

SCLC: Small cell lung cancer
TTPD: Time to progressive disease
CSS: Chemotherapy-specific survival
PD: Progressive disease
OS: Overall survival
CR: Complete response
PR: Partial response
SD: Stable disease
ORR: Overall response rate.
