# Supplementary material for: Lesser Toxicities of Belotecan in Patients with Small Cell Lung Cancer: A Retrospective Single-Center Study of Camptothecin Analogs
Source: Can Respir J. 2016 Nov 27;2016:3576201. doi: 10.1155/2016/3576201 (PMC5149640; doi:10.1155/2016/3576201)
Supplement: Supplementary file 1 — Kaplan-Meier survival analysis [file 3576201.f1.pdf]

<Supplemental appendix>

Figure legend

Figure 1. Kaplan-Meier survival analysis using the log-rank test for (a) time to progressive disease, (b) chemotherapy-specific survival, and (c) overall survival for belotecan or topotecan monotherapy in patients with small cell lung cancer.

Figure 1(a)

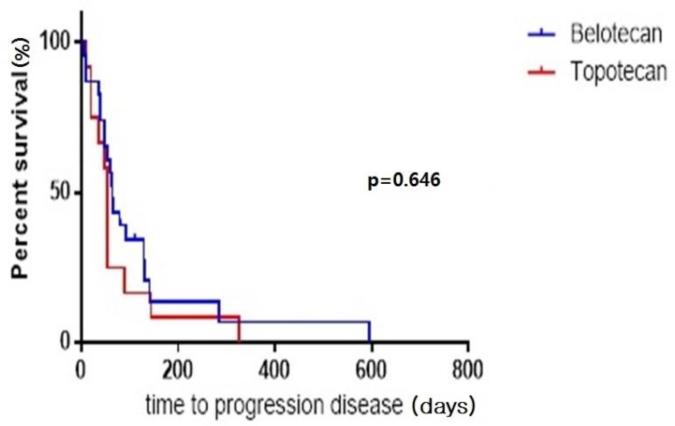

Figure 1(b)

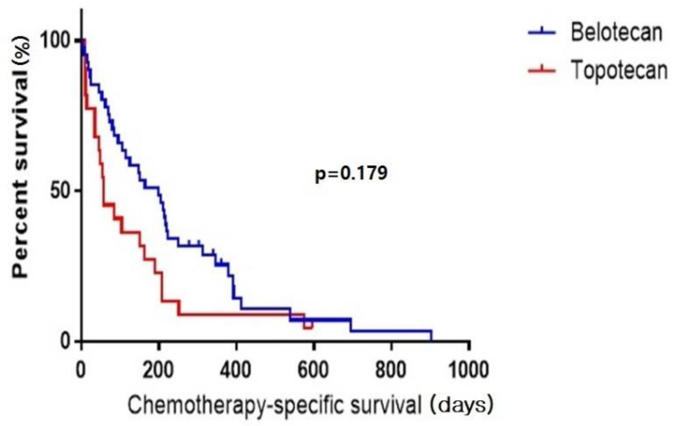

Figure 1(c)

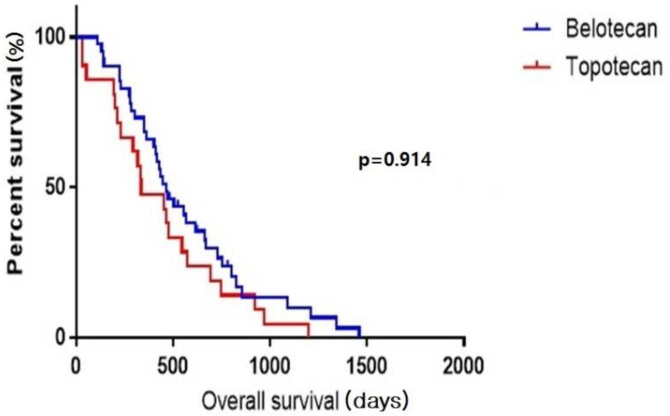
Belotecan
Topotecan
p=0.914
Percent survival(%)
100
50
0
0
500
1000
1500
2000
Overall survival (days)
